# The intangible costs of overweight and obesity in Germany

**DOI:** 10.1186/s13561-023-00426-x

**Published:** 2023-02-21

**Authors:** Fan Meng, Peng Nie, Alfonso Sousa-Poza

**Affiliations:** 1 Institute for Health Care & Public Management, University of Hohenheim, 70599 Stuttgart, Germany; 2 School of Economics and Finance, Xi’an Jiaotong University, Xi’an, 710061 China; 3 IZA, Bonn, Germany; 4 Health Econometrics and Data Group, University of York, York, UK

**Keywords:** Intangible costs, Obesity, Overweight, Germany, I10, I12, R21

## Abstract

**Background:**

Previous literature documents the direct and indirect economic costs of obesity, yet none has attempted to quantify the intangible costs of obesity. This study focuses on quantifying the intangible costs of one unit body mass index (BMI) increase and being overweight and obese in Germany.

**Methods:**

By applying a life satisfaction-based compensation value analysis to 2002–2018 German Socio-Economic Panel Survey data for adults aged 18–65, the intangible costs of overweight and obesity are estimated. We apply individual income as a reference for estimating the value of the loss of subjective well-being due to overweight and obesity.

**Results:**

The intangible costs of overweight and obesity in 2018 amount to 42,450 and 13,853 euros, respectively. A one unit increase in BMI induced a 2553 euros annual well-being loss in the overweight and obese relative to those of normal weight. When extrapolated to the entire country, this figure represents approximately 4.3 billion euros, an intangible cost of obesity similar in magnitude to the direct and indirect costs documented in other studies for Germany. These losses, our analysis reveals, have remained remarkably stable since 2002.

**Conclusions:**

Our results underscore how existing research into obesity’s economic toll may underestimate its true costs, and they strongly imply that if obesity interventions took the intangible costs of obesity into account, the economic benefits would be considerably larger.

**Supplementary Information:**

The online version contains supplementary material available at 10.1186/s13561-023-00426-x.

## Introduction

As regards weight statistics, Germany currently ranks in the upper middle among OECD countries, with about two-thirds of men and half of women being overweight, a quarter of all Germans being obese [1], and an obesity prevalence double the 2000 rate of 12% [2, 3]. As a risk factor for a variety of chronic illnesses − including type 2 diabetes mellitus [4], cardiovascular disease [5, 6], and cancer [7] – obesity raises the risk of premature death [8, 9] and poses a serious challenge for health systems in Germany and across the globe. Hence, the World Health Organization [10] formulated a goal of no further increase in obesity rates between 2010 and 2025, a goal also adopted as part of the German Federal Government’s 2021 Sustainability Development Strategy.

Given these obesity-related health concerns and corresponding health policy measures, it is unsurprising that numerous studies document the obesity-related economic costs to Germany [11–17], which one of the most comprehensive calculates at around 63 billion euros annually as of 2012 [16]. Whereas about half this sum (€29.39 billion) refers to directly attributable (medical and nonmedical) costs such as diagnosis, treatment, medication, prevention, nursing care, rehabilitation, and accidents, the other half reflects indirect costs associated with productivity loss, including obesity-related absenteeism, unemployment, premature retirement, or premature death. Obesity can also give rise to “intangible” costs not reflected by market-valued transactions but rather directly associated with the pain of losing subjective well-being (SWB) [16] via either obesity-related comorbidities or bullying, stigmatization, and discrimination.

Yet although most research on the cost of obesity acknowledges the existence and importance of intangible costs, we find no study that comprehensively calculates their economic toll. For instance, even though Effertz et al. [16] partially consider intangible costs by using physicians’ ICD coding to estimate the probability of obesity-related pain, their analysis, as the authors acknowledge, provides only rough insights into pain frequency during the obese individual’s life cycle with no assessment of its monetary value. Nor does it capture any of the loss of well-being caused by discrimination or bullying. This research void is rather surprising given not only the potential economic significance of such intangible costs but also obesity’s well-documented negative effects on SWB [18–20], often through stigmatization and discrimination [21]. For example, in the US, obese individuals earn about 10% less than their healthy weight counterparts even with productivity controlled for [22] and may even be blatantly dehumanized [23].

The most obvious reason for this dearth is the perceived inability to evaluate associated losses of well-being as market transactions, even though valuing such intangible costs (or well-being losses) has a long tradition in economic studies on pollution [24, 25], fear of crime [26], commuting [27], and overeducation [28]. In these instances, researchers commonly use a life satisfaction-based compensation value (i.e., shadow price) approach to estimate intangible cost. The researcher assigns a monetary value to the intangible losses by calculating how much income is needed to compensate them. This is equivalent to computing the marginal rate of substitution between income and the negative intangible effect. In this present study, therefore, we apply this approach to 2002–2018 German Socio-Economic Panel (SOEP) data to produce what we believe to be the first estimation of obesity’s intangible costs in Germany. Analyzing these costs over such a long period is especially useful because we currently have no a priori knowledge on how the marginal rate of substitution between income and obesity has evolved. If, for instance, the marginal utility of income and the marginal (dis)utility of obesity are not constant across time, then, all else being equal, decreasing stigmatization as obesity levels rise could reduce the latter’s disutility and lower its intangible costs.

### The conceptual framework: life satisfaction approach

Our life satisfaction compensation approach [24] calculates the monetary value of three bodyweight measures − BMI, overweight, and obesity − based on the amount of net annual income needed to compensate the life satisfaction lost from a one-unit increase in BMI or overweight/obesity relative to normal weight. After first defining utility as

1 $$U=U\left(B,Y\right)$$

where *B* is individual bodyweight status and *Y* is income, we obtain total differentiation by setting *dU* = 0, which yields

2 $$dU={MU}_B\cdot dB+{MU}_Y\cdot dY=0$$

Sorting gives

3 $$dY/ dB=-M{U}_B/M{U}_Y$$

Next, using a quasi-linear utility function of the following form,

4 $$U=\beta B+\delta lnY$$

we obtain

5 $${MU}_B=\beta$$

6 $${MU}_Y=\delta /Y$$

We can then express the income required to compensate an increase in obesity as follows:

7 $$dY/ dB=-\beta Y/\delta$$

or

8 $$Cost= YI$$

where *I* denotes the negative quotient of *β* and *δ*. Eq. 7 allows us to calculate the marginal rate of substitution between income (*Y*) and the bodyweight (*B*). Hence, we can estimate the monetary value of compensation for an additional unit of BMI while also quantifying the costs of overweight or obesity relative to normal weight when given corresponding *β*, *δ*, and income (*Y*). We will employ different empirical strategies to estimate the coefficients *β* and *δ* and measure the estimated cost based on them.

### Data and methods

#### Survey and sample

We draw our data from the German Socio-Economic Panel (SOEP) version 35 (10.5684/soep-core.v35), a nationally representative longitudinal survey administered annually since 1984 by the German Institute for Economic Research. Interviews are currently conducted via computer-assisted personal interviews (CAPI) to approximately 30,000 of about 15,000 households [29]. Using the latest available wave (2018), we restrict our sample to adults aged 18–65 and exclude respondents who are underweight, without positive income, or have implausible BMI values (BMI > 60) [30, 31] for a final 2018 sample of 11,407 respondents.^1^ In addition to providing the most recent estimates of obesity’s intangible costs, we also examine their evolution by analyzing nine survey waves that include information on individual weight and height (i.e., 2002, 2004, 2006, 2008, 2010, 2012, 2014, 2016, and 2018) for a combined sample of 33,425 individuals and 100,369 observations.

#### Variables

##### SWB measure

Our key proxy of SWB is life satisfaction, whose measure we derive from responses to the question “How satisfied are you with your life, all things considered?” ranked on an 11-point Likert scale from 0 = completely dissatisfied to 10 = completely satisfied.

##### Bodyweight measures

We calculate BMI (kg/m^2^) based on self-reported height and weight, with normal weight defined as a BMI between 18.5 and 25 kg/m^2^. Our bodyweight measures are overweight (BMI 25–30 kg/m^2^) and general obesity (BMI ≥ 30 kg/m^2^).

##### Income

Because our life satisfaction approach requires an income measure in addition to SWB and bodyweight, we include net annual individual income (in euros) calculated as net monthly income (i.e., after deduction of taxes and social security/unemployment/health insurance) multiplied by 12. When using multiple years, we deflate income to 2018 prices using the Consumer Price Index (CPI) [32].

##### Individual and household characteristics

Our life satisfaction models include the standard controls [25, 26] for individual demographic and socioeconomic characteristics, including age, age squared, gender (1 = female, 0 = male), education, and marital status. Education is measured by years of schooling ranging from 7 to 18. Marital status is first measured on a 5-point scale of 1 = married, 2 = single, 3 = widowed, 4 = divorced, and 5 = separated, and then recoded as a binary dummy variable with 1 = married and 0 otherwise. Because homeowners tend to have a higher level of life satisfaction than tenants [33, 34], the household characteristics include homeownership as well as number of children, with homeownership being a binary variable equal to 1 if the respondent owns his/her dwelling (0 otherwise).

### Estimation strategies

In order to estimate the coefficients *β* and *δ* in Eq. 7, we estimate a regression of the following form:

9 $${SWB}_i={\alpha}_1+{\beta}_1{BMI}_i+{\delta}_1\ln \left({income}_i\right)+{\gamma}_1{X}_i+{\rho}_1{F}_i+{\varepsilon}_i$$

where *SWB*_*i*_, *BMI*_*i*_, and *ln*(*income*_*i*_) denote individual *i* ’s life satisfaction, BMI, and translog net income, respectively , *X*_*i*_ is a vector of individual characteristics, and *F*_*i*_, a vector of household characteristics. Here, the individual characteristics are age, age squared, gender, education, and marital status; and the household characteristics are homeownership and the number of children. *β*_1_ captures the association between each individuals’ BMI and SWB, with *ε*_*i*_ as the error term. We use both an ordinary least squares (OLS) and an ordered logit model to estimate this equation. Although the 11-point scaling of the life satisfaction measure may suggest the use of latent variable estimation, the bias from the OLS approach used most commonly in the literature [27, 35] is small enough [36]. Note that this regression is only run for overweight and obese individuals as it can be plausibly assumed that increases in BMI among normal individuals would not incur any costs.

Using similar specifications to those in Eq. 9, we estimate the model below to analyze the association of SWB with overweight and obesity:

10 $${SWB}_i={\alpha}_2+{\beta}_2{overweight}_i+{\beta}_3{obese}_i+{\delta}_2\mathit{\ln}\left({income}_i\right)+{\gamma}_2{X}_i+{\rho}_2{F}_i+{u}_i$$

where *overweight*_*i*_ and *obese*_*i*_ are binary dummies indicating individual *i* ’s weight status, with normal weight as the reference. We also compare the intangible costs of these two groups in an additional regression using only overweight and obese individuals:

11 $${SWB}_i={\alpha}_3+{\beta}_4{obese}_i+{\delta}_3\mathit{\ln}\left({income}_i\right)+{\gamma}_3{X}_i+{\rho}_3{F}_i+{v}_i$$

where *obese*_*i*_ denotes whether individual *i* is obese or not, with overweight as the reference.

Lastly, using Eqs. 9 and 10, we estimate the intangible costs between 2002 and 2018 to assess their dynamics.

We run a number of robustness tests in order to check for the three most common sources of bias: measurement error, omitted variables, and reverse causality. The first may stem from our use of self-reported income, weight, and height measures, which could result in underestimation of actual earnings and BMI [37]. Omitted variable bias could arise if certain unobserved factors affect individual BMI and SWB simultaneously; for example, if personality traits that affect obesity also influence SWB [38]. The final concern, reverse causality, may occur if SWB influences obesity (e.g., through eating habits) as reflected by happier individuals in some societies tending toward higher BMIs [39].

Our primary approach to addressing these potential biases is to adopt an instrument variable (IV) model capable of handling the endogeneity problem, one whose instrument must fulfill the exclusion restriction. Given the absence of any obvious exogenous IV − and having confirmed the error term’s heteroskedasticity via a Breusch-Pagan test [40]− we adopt Lewbel’s (2012) 2SLS approach, which requires heteroskedasticity as a precondition for identification. Both Lewbel [41] and Mishra and Smyth [42] confirm that, given a suitable external IV, this method yields comparable results to those from a conventional external IV while also offering the advantage of combinability with a standard excluded instrument [43–45].^2^ The approach has thus produced useful insights not only in research on mental health and SWB [46, 47] but also in diverse fields of economics [42, 48].

We first assume a triangular system in Eqs. 12 and 13 where *SWB*_*i*_ and *BMI*_*i*_ are endogenous, *X*^′^ is a vector of exogenous covariates, and *ϵ*_1_ and *ϵ*_2_ are unobserved errors that may correlate with each other. As in a standard IV approach, the exogeneity assumption that *E*(*ϵX*) = 0 and *E*(*XX*^′^) is satisfied, and *E*(*XX*^′^) is nonsingular. The essential extra conditions of the Lewbel [41] estimator are that *Cov*(*Z*, *ϵ*_1_*ϵ*_2_) = 0 and $Cov\left(Z,{\epsilon}_{2}^{2}\right)\ne 0$, where *Z* ⊆ *X*. Here, the instruments are *X* and $\left(Z-\overline{Z}\right)\hat{\epsilon_2}$, where $\overline{Z}$ is the mean of *Z*:

12 $${SWB}_i=a+{\beta}_5{X}_i^{\prime }+{\beta}_6{BMI}_i+{\epsilon}_1$$

13 $${BMI}_i=b+{\beta}_7{X}_i^{\prime }+{\epsilon}_2$$

We treat income as exogenous when applying the Lewbel [41] IV approach to BMI to verify the causal relation between bodyweight and life satisfaction. Although income may also be endogenous, the condition of validity for more than one endogenous regressor has not been demonstrated [45].

We further confirm the robustness of our results by first using split analyses by income tercile to check the stability and magnitude of our primary findings in different income groups. In doing so, we ensure as large a sample as possible by estimating Eq. 9 with pooled cross-sectional data (2002–2018) and include year dummies (with 2002 as the reference year). We also partially account for omitted variables bias (caused by time-invariant variables) by using 2016 and 2018 SOEP data to estimate the following fixed effects (FE) model:

14 $${SWB}_{it}={\alpha}_6+{\beta}_8{BMI}_{it}+{\delta}_6\mathit{\ln}\left({income}_{it}\right)+{\gamma}_6{X}_{it}+{\rho}_6{F}_{it}+{\omega}_i+{\varepsilon}_{it}$$

where *ω*_*i*_ captures unobservable time-invariant individual effects, *X*_*it*_ (*F*_*it*_) is a vector of individual *i*’s time-variant (household) characteristics in period t, and *ε*_*it*_ is the error term.

## Results

### Descriptive statistics

As Table 1 shows, the average values of life satisfaction and BMI for our sample are 7.527 and 26.505 kg/m^2^ in 2018, respectively, with over half of the respondents being overweight or obese (cf. Schienkiewitz et al. [3]). As in Biewen et al. [49], the mean annual income after tax is approximately 22,899 euros. The gender distribution is almost equal (50.2% female), with an average age around 44. A majority (61%) is married with approximately 13 years of education.^3^ For respondents with a BMI between 18.5 and 60 kg/m^2^ (25 and 60 kg/m^2^), however, the BMI values increase from 25.4 (28.6) in 2002 to 26.6 (29.8) in 2018, suggesting an increasing trend in mean BMI among German adults (see Additional file 1: Table A2, Panels A and B).

**Table 1:** Descriptive statistics: SOEP 2018

Variables	Obs.	Mean	S.D.
Life satisfaction	11,407	7.527	1.535
Body mass index (BMI, kg/m^2^)	11,407	26.505	4.974
Obesity^a^	11,407	0.198	0.399
Overweight^a^	11,407	0.358	0.480
Normal weight^a^	11,407	0.444	0.497
Net annual income (euros)	11,407	22,898.74	22,228.35
Age	11,407	44.087	11.579
Female^a^	11,407	0.502	0.500
Married^a^	11,407	0.614	0.487
Years of education	11,407	12.602	2.841
Number of children in the household	11,407	0.913	1.122
Homeowner^a^	11,407	0.483	0.500

BMI means body mass index, which is defined as height (in m) divided by weight (in kg) squared. The measures of obesity, overweight, and normal weight are based on BMI, which is defined as obesity (BMI ≥ 30), overweight (25 ≤ BMI < 30), and normal weight (18.5 ≤ BMI < 25)

^a^Dummy variables

### OLS and ordered logit estimates

Our OLS and ordered logit analyses of the intangible costs of BMI, overweight, and obesity (see Table 2) pinpoint three key findings: First, relative to normal weight, overweight and obesity have intangible costs in 2018 of 13,853 and 42,450 euros (OLS) or 17,868 and 45,502 euros (ordered logit), respectively (see columns 1 & 2, Panel A), implying that overweight and obese individuals suffer from larger well-being losses than those of normal weight. At the same time, a one-unit additional increase in BMI among the overweight and obese resulted in 2553 (2562) euros of well-being loss, while obesity had an annual intangible cost of 23,261 (24,294) euros relative to overweight (see columns 1 & 2, Panel B).

**Table 2:** Yearly intangible costs of BMI, overweight, and obesity: SOEP 2018

	OLS (1)	Ordered logit (2)
**Panel A: 18.5** ≤ **BMI** ≤ **60**		
Cost of overweight	13,853**	17,868**
95% CI	[1988; 25,717]	[5823; 29,913]
Cost of obesity	42,450***	45,502***
95% CI	[21,281; 63,618]	[24,430; 64,575]
Observations	11,407	11,407
**Panel B: 25** ≤ **BMI** ≤ **60**		
Cost of BMI	2553***	2562***
95% CI	[902; 4204]	[989; 4134]
Cost of obesity	23,261***	24,294***
95% CI	[8458; 38,065]	[9388; 39,201]
Observations	6347	6347

*BMI* body mass index, defined as height (in m) divided by weight (in kg) squared. Costs are in euros. The overweight, BMI, and obesity costs in Panels A and B are calculated based on Eqs. 9, 10 and 11 respectively. The 95% confidence intervals, given in brackets, are calculated using Fieller’s theorem

Significance levels are shown as *** *p* < 0.01, ** *p* < 0.05, * *p* < 0.1

### Intangible costs of bodyweight 2002–2018

**Fig. 1:**
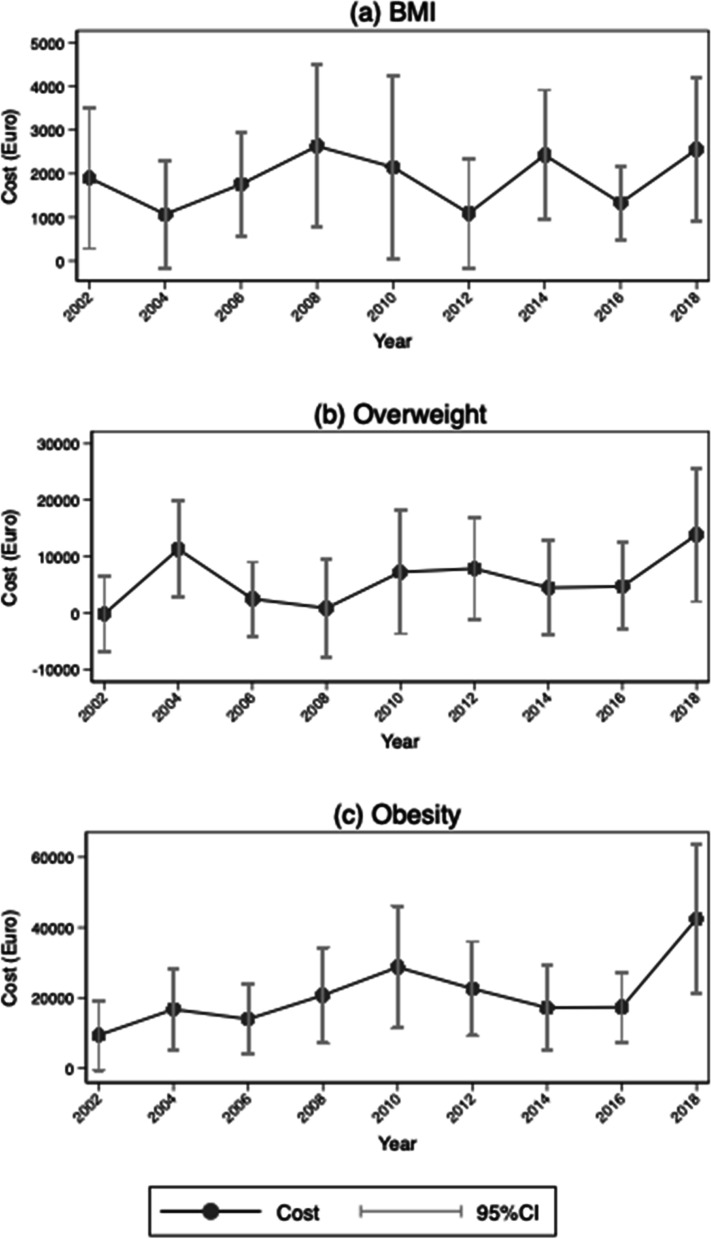
Trends in the intangible costs of BMI, overweight, and obesity: SOEP 2002–2018. **a** the trend in intangible costs for BMI; **b** and **c** the trends for overweight and obesity, respectively. BMI = body mass index, defined as height (in m) divided by weight (in kg) squared. Obesity = BMI ≥ 30; overweight = 25 ≤ BMI < 30. Confidence intervals are calculated using Fieller’s theorem

**Fig. 2:**
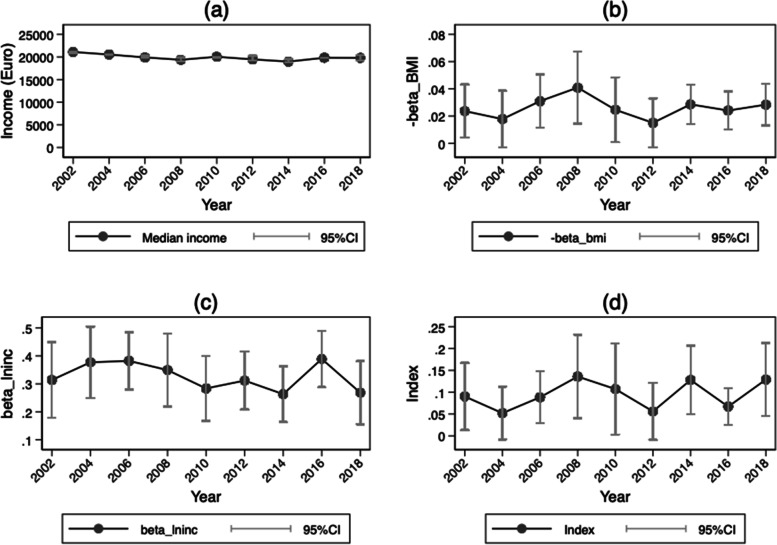
Trends in the components of the intangible costs of BMI: SOEP 2002–2018. **a** median annual net income per year in 2018 euros; **b** and **c** the coefficients of BMI and income, respectively, based on Eq. 9; **d** the trend in the index, which denotes the negative division of the coefficient of BMI and income. Confidence intervals are calculated using Fieller’s theorem

Although graphing the trends in obesity-attributable intangible costs from 2002 to 2018 suggests a slight increase in obesity (Fig. 1c), it reveals no general pattern.^4^ Hence, given our estimations’ reliance on the marginal effects of obesity and income on life satisfaction, we strive to expand our understanding of the cost dynamics by mapping these key parameters. As Fig. 2 shows, the trends for income and the estimated coefficients of BMI, income, and Eq. 9 all remain remarkably stable across time. The trends for the estimated coefficients of overweight and obesity are shown in Additional file 1: Figs. A1 and A2, respectively.

### Robustness and heterogeneous analysis

When we compile the different intangible costs of BMI by income terciles based on pooled OLS and ordered logit estimates (see Table 3), we find that, compared to the low- and middle-level income groups, the high-level income group experiences the largest BMI-related loss of well-being irrespective of estimation type.^5^ This finding implies that the richest may suffer from the largest intangible costs attributable to an additional BMI increment.

**Table 3:** OLS/ordered logit estimates of BMI’s intangible costs by income tertile: SOEP 2002–2018

	Low (1)	Middle (2)	High (3)
**Panel A: OLS**			
Costs of BMI	1390***	775***	2169***
95% CI	[560; 2221]	[296; 1253]	[1405; 2933]
Observations	15,274	17,609	19,859
**Panel B: Ordered logit**			
Costs of BMI	1795***	807***	2014***
95% CI	[666; 2924]	[324; 1291]	[1331; 2696]
Observations	15,274	17,609	19,859

*BMI* body mass index, defined as height (in m) divided by weight (in kg) squared. Costs are in euros. The 95% confidence intervals, given in brackets, are calculated using Fieller’s theorem

Significance levels are shown as *** *p* < 0.01, ** *p* < 0.05, * *p* < 0.1

Given the potential for BMI endogeneity, in this analysis, we employ both FE and Lewbel IV estimations, both of which corroborate the significant negative association between increased BMI and life satisfaction (see Table 4). Not only does a Breusch-Pagan test verify the appropriateness of the Lewbel IV method by confirming the existence of heteroskedasticity, but the first-stage *F* statistics, which greatly exceed 10, imply no weak instruments, while the Hanson *J* test affirms the exogeneity of our IVs. According to the FE estimation, the BMI-related intangible cost is 3229 euros, while that from the Lewbel IV is a lower 2590 euros.

**Table 4:** Fixed effects/Lewbel IV estimates of BMI on life satisfaction: SOEP 2016, 2018

	FE^a^ (1)	Lewbel IV^b^ (2)
BMI	−0.024**	−0.027**
	(0.011)	(0.014)
Costs	3229**	2590**
95% CI	[278; 6180]	[14; 5165]
Controls	Yes	Yes
Under identification test		< 0.001
Weak instrument (*F*-statistic)		53.061
Hansen J statistic (*p*-value)		0.894
Observations	13,379	6315

Dependent variable = life satisfaction. BMI = body mass index, defined as height (in m) divided by weight (in kg) squared. This analysis includes samples with BMI ≥ 25. Costs are in euros. The FE model controls for translog income, age, age squared, years of education, number of children, and homeownership, while the Lewbel IV adds in gender and marital status but omits age squared. Standard errors in parentheses; 95% confidence intervals (CI) in brackets

Significance levels are shown as *** *p* < 0.01, ** *p* < 0.05, * *p* < 0.1

^a^ Based on 2016 and 2018 data

^b^ Based on 2018 data only

## Discussion and conclusions

A large international body of literature documents the economic costs of obesity (e.g. [50]), which, although the estimates vary considerably depending on data and methods, are universally agreed to be substantial. In Germany, for example, the annual economic costs can range between 9.87 billion and 63.04 billion euros [12, 16]. Yet all these studies, while acknowledging the existence of obesity’s intangible costs, make no attempt to quantify them, focusing only on the direct and indirect expenses. This failure is surprising not only because of the widely documented obesity-SWB link [18–20] and obesity stigmatization [51], but because a long tradition of intangible cost estimation in several economics field (e.g., transport, environmental, and public economics) has furnished a viable, but as yet unused, method for measuring obesity’s intangible costs.

In this paper, we adopt this life satisfaction approach to estimate the intangible costs of obesity in Germany using rich longitudinal SOEP data. According to our results, not only did the overweight and obese incur 2018 costs of 13,853 and 42,450 euros, respectively, relative to normal-weight individuals, but a one-unit increase in BMI among these groups induced a 2553 euro loss in well-being, which extrapolates to a national cost of approximately 4.3 billion euros.^6^ To assess the stability of these intangible costs − which, unlike direct and indirect costs, are by definition a reflection of societal views on obesity − we also estimate them longitudinally (2002–2018), probing for changing discrimination and stigmatization patterns over time as perceptions of ideal body composition vary [52, 53]. In the US, for example, an increasing incidence of obesity has raised American notions of an ideal weight until a growing number of obese individuals see themselves as normal. Our results for Germany, however, show no clear trend. Rather, the intangible costs of obesity remain remarkably stable across time, with neither its effect on SWB nor its effect on income changing noticeably over the past two decades despite a large concurrent increase in obesity rates. This result is interesting as it shows that even over a relatively long time period, the marginal utility of income and the marginal disutility of obesity remain quite constant. In the case of income, this may not be too surprising as real income levels have not changed much. However, in the case of obesity, we have witnessed a large increase in its prevalence, yet no change in the marginal disutility. One plausible assumption would be that as obesity rates rise, a society not only becomes more tolerant of obesity, but also may change its perception about an ideal body image. One possible reason for not observing such an assumed change in the marginal disutility of obesity is that our 16-year analysis may be too short to capture changes in society’s perception regarding obesity. In this context it is worth noting that the change of ideal body image in the United States is smaller than the actual change of average weight [53]. Considering the slower rise of obesity in Germany that in the United States, one can assume that perceptions in Germany are changing slowly.

Our study is of course subject to certain limitations; in particular, the relatively large 95% confidence intervals for the estimated income and obesity coefficients in the SWB regressions, which show obesity costs ranging from approximately 21,000 to 64,000 euros. Not only are such large confidence intervals commonplace in life satisfaction-based analyses,^7^ however (see, e.g., for overeducation [28] and drought [54]), but even the lowermost bounds of these intervals mark the intangible costs as substantial. We also recognize the life satisfaction approach’s inherent susceptibility to endogeneity issues as a result of the pertinent explanatory variables (in our case, obesity) being so often endogenous. In our study, however, unlike most others, we not only acknowledge obesity’s endogeneity − and particularly the resulting risk from reverse causality − but perform a robustness check using a heteroskedasticity-based IV estimator. Despite the challenge of controlling simultaneously for several potentially endogenous variables (most notably income, but also obesity and overweight), our IV results with BMI as the sole instrument support our cross-sectional results. As regards the additional concern of measurement errors from the self-reporting of height and weight [55, 56], the SOEP is the only available nationally representative dataset that provides measures of BMI covering a time-span of nearly two decades. Besides the widespread use of the SOEP obesity data [13, 57], there is also some evidence that such self-reports are reasonably accurate [58].

Despite these limitations, our results underscore how significantly existing research into obesity’s economic toll may underestimate its true costs, an especially important caveat for the myriad evaluations of obesity-related policy and environmental interventions [59]. Our findings strongly imply that if these interventions took intangible costs into account, the economic benefits would be considerably larger. Yet to date, economic evaluations of obesity interventions measure outcomes only in health-related terms (either quality-adjusted life years (QALYs), disease-adjusted life years (DALYs), or natural health units), thereby ignoring the impact of overweight and obesity on general well-being beyond health-related measures of quality of life [60]. Yet had the cost benefit analysis that found Australia’s “reformulation in response to the Health Star Rating system” and “community-based interventions” to combat obesity [61] to be cost ineffective taken into account intangible costs of at least similar magnitude to the direct and indirect costs, it might have reached the opposite conclusion. Given the global obesity pandemic, accurate assessment of obesity’s true cost to society is vital, including consideration of its intangible costs in any intervention-related decision [61, 62].

## Supplementary Information

**Additional file 1: Table A1.** Descriptive statistics for adults aged 18–65: SOEP 2002–2018. **Table A2.** Descriptive statistics of BMI: SOEP 2002–2018. **Table A3.** OLS/ordered logit estimates of bodyweight on life satisfaction: SOEP 2018. **Table A4.** OLS estimates of BMI on life satisfaction: SOEP 2002–2018. **Table A5.** OLS estimates of overweight and obesity on life satisfaction: SOEP 2002–2018. **Table A6.** OLS/ordered logit estimates of BMI on life satisfaction by different income levels: SOEP 2002–2018. **Figure A1.** Trends in the components of the intangible cost of overweight. **Figure A2.** Trends in the components of the intangible cost of obesity.

## Data Availability

The data underlying the results presented in the study are available from https://www.diw.de/.

## Abbreviations

BMI: Body mass index
SWB: Subjective well-being
SOEP: Socio-Economic Panel
CAPI: Computer-assisted personal interviews
CPI: Consumer Price Index
OLS: Ordinary least squares
IV: Instrument variable
FE: Fixed effects
QALYs: Quality-adjusted life years
DALYs: Disease-adjusted life years

## Footnotes

1 The number of respondents before exclusion is 11,984 in 2018.

2 For this study, we implement the Lewbel [41] IV approach using use the Stata “ivreg2h” syntax (see Baum & Schaffer [63]).

3 The descriptive statistics for the pooled data are given in Additional file 1: Table A1.

4 Additional file 1: Tables A4 and A5 report the regression results for BMI and overweight/obesity based on Eqs 9 and 10, respectively.

5 Detailed results are given in Additional file 1: Table A6.

6 We calculate this extrapolation by first multiplying the average increase in BMI from 2016 to 2018 by 2553 euros and by the number of obese and overweight individuals in 2018 and then dividing the result by 2 to derive an annual value. This extrapolation thus represents the additional costs incurred in one year by the average rise in BMI, which our IV estimates put at 4.4 billion.

7 Large confidence intervals are a common problem when using Fieller’s theorem to calculate the confidence interval for the ratio of two coefficients. Since the two coefficients have different standard errors, Fieller’s method constructs a normal distribution by using a linear combination of that ratio and the mean of the two coefficients [64]. The variance of this new normal distribution is larger than the variance of the original two coefficients. The estimated confidence interval for the ratio can be obtained by constructing a variable with a chi-squared distribution with 1 degree of freedom and solving a quadratic inequality. This process may further amplify the confidence interval. Detailed regression results are reported in Additional file 1: Table A3.
