# Supplementary material for: The intangible costs of overweight and obesity in Germany
Source: Health Econ Rev. 2023 Feb 21;13:14. doi: 10.1186/s13561-023-00426-x (PMC9942367; doi:10.1186/s13561-023-00426-x)
Supplement: Supplementary file 1 — Additional file 1: Table A1. Descriptive statistics for adults aged 18–65: SOEP 2002–2018. Table A2. Descriptive statistics of BMI: SOEP 2002–2018. Table A3. OLS/ordered logit estimates of bodyweight on life satisfaction: SOEP 2018. Table A4. OLS estimates of BMI on life satisfaction: SOEP 2002–2018. Table A5. OLS estimates of overweight and obesity on life satisfaction: SOEP 2002–2018. Table A6. OLS/ordered logit estimates of BMI on life satisfaction by different income levels: SOEP 2002–2018. Figure A1. Trends in the components of the intangible cost of overweight. Figure A2. Trends in the components of the intangible cost of obesity. [file 13561_2023_426_MOESM1_ESM.docx]

**Additional file**

| **Table A1** Descriptive statistics for adults aged 18-65: SOEP 2002-2018 | | | |
| --- | --- | --- | --- |
| Variables | Obs. | Mean | S.D. |
| Life satisfaction (0-10) | 100,369 | 7.261 | 1.595 |
| Body mass index (kg/m^2^) | 100,369 | 25.961 | 4.514 |
| Obesity^a^ | 100,369 | 0.162 | 0.369 |
| Overweight^a^ | 100,369 | 0.363 | 0.481 |
| Normal weight^a^ | 100,369 | 0.475 | 0.499 |
| Net annual income | 100,369 | 23177.280 | 20533.620 |
| Age | 100,369 | 43.171 | 11.356 |
| Female^a^ | 100,369 | 0.485 | 0.500 |
| Married^a^ | 100,369 | 0.621 | 0.485 |
| Years of education | 100,369 | 12.689 | 2.752 |
| Number of children in household | 100,369 | 0.741 | 1.013 |
| Homeowner^a^ | 100,369 | 0.526 | 0.499 |
| ^a^ Dummy variables  Notes: BMI = body mass index, defined as height (in m) divided by weight (in kg) squared. Obesity = BMI≥30; overweight = 25≤BMI<30; normal weight = 18.5≤BMI<25. Net annual income is in euros. | | | |

| **Table A2** Descriptive statistics of BMI: SOEP 2002-2018 | | | | | |
| --- | --- | --- | --- | --- | --- |
| Survey years | Weighted obs. | Mean | SD | Min. | Max. |
| Panel A: 18.5≤BMI≤60 | | | | | |
| 2002 | 30,225,965 | 25.402 | 4.021 | 18.508 | 56.495 |
| 2004 | 30,354,902 | 25.557 | 4.127 | 18.508 | 57.099 |
| 2006 | 30,500,125 | 25.744 | 4.326 | 18.508 | 59.515 |
| 2008 | 32,661,480 | 25.983 | 4.452 | 18.513 | 59.028 |
| 2010 | 28,832,815 | 26.220 | 4.639 | 18.508 | 56.811 |
| 2012 | 27,528,187 | 26.217 | 4.645 | 18.508 | 58.280 |
| 2014 | 33,369,471 | 26.250 | 4.756 | 18.508 | 58.228 |
| 2016 | 33,595,886 | 26.435 | 4.813 | 18.508 | 58.770 |
| 2018 | 27,174,173 | 26.627 | 5.021 | 18.508 | 58.770 |
| Panel B: 25≤BMI≤60 | | | | | |
| 2002 | 14,459,108 | 28.624 | 3.278 | 25 | 56.495 |
| 2004 | 15,071,400 | 28.714 | 3.407 | 25 | 57.099 |
| 2006 | 15,644,538 | 28.902 | 3.641 | 25 | 59.515 |
| 2008 | 17,321,454 | 29.097 | 3.775 | 25 | 59.028 |
| 2010 | 15,732,495 | 29.337 | 3.965 | 25 | 56.811 |
| 2012 | 15,099,007 | 29.322 | 3.962 | 25 | 58.280 |
| 2014 | 18,066,621 | 29.464 | 4.112 | 25 | 58.228 |
| 2016 | 18,771,482 | 29.568 | 4.127 | 25 | 58.770 |
| 2018 | 15,338,314 | 29.788 | 4.424 | 25 | 58.770 |
| Note: BMI = body mass index, defined as height (in m) divided by weight (in kg) squared. These results have sampling weights applied. | | | | | |

| **Table A3** OLS/ordered logit estimates of bodyweight on life satisfaction: SOEP 2018 | | | | | | | |
| --- | --- | --- | --- | --- | --- | --- | --- |
|  | BMI≥18.5 | |  | BMI≥25 | | | |
|  | (1) | (2) |  | (3) | (4) | (5) | (6) |
|  | OLS | Ordered logit |  | OLS | OLS | Ordered logit | Ordered logit |
| Overweight^a^ | -0.127** | -0.206*** |  |  |  |  |  |
|  | (0.054) | (0.066) |  |  |  |  |  |
| Obesity^a^ | -0.378*** | -0.510*** |  |  | -0.257*** |  | -0.320*** |
|  | (0.067) | (0.080) |  |  | (0.067) |  | (0.081) |
| BMI (kg/m^2^) |  |  |  | -0.028*** |  | -0.034*** |  |
|  |  |  |  | (0.008) |  | (0.009) |  |
| Ln(income) | 0.207*** | 0.259*** |  | 0.268*** | 0.269*** | 0.313*** | 0.314*** |
|  | (0.041) | (0.047) |  | (0.058) | (0.058) | (0.066) | (0.066) |
| Age | -0.040** | -0.062*** |  | -0.041* | -0.043* | -0.056** | -0.059** |
|  | (0.016) | (0.020) |  | (0.023) | (0.023) | (0.028) | (0.028) |
| Age squared | 0.000* | 0.001** |  | 0.000 | 0.000 | 0.001* | 0.001* |
|  | (0.000) | (0.000) |  | (0.000) | (0.000) | (0.000) | (0.000) |
| Female^a^ | 0.156*** | 0.207*** |  | 0.121 | 0.113 | 0.138 | 0.129 |
|  | (0.054) | (0.063) |  | (0.076) | (0.075) | (0.089) | (0.088) |
| Years of education | 0.013 | 0.012 |  | -0.008 | -0.007 | -0.009 | -0.008 |
|  | (0.009) | (0.011) |  | (0.013) | (0.013) | (0.015) | (0.015) |
| Married^a^ | 0.480*** | 0.562*** |  | 0.564*** | 0.563*** | 0.676*** | 0.672*** |
|  | (0.055) | (0.067) |  | (0.073) | (0.073) | (0.089) | (0.089) |
| Number of children | 0.047* | 0.083** |  | 0.033 | 0.039 | 0.072 | 0.080* |
|  | (0.026) | (0.034) |  | (0.034) | (0.034) | (0.047) | (0.047) |
| Homeowner^a^ | 0.118** | 0.133** |  | 0.060 | 0.053 | 0.037 | 0.025 |
|  | (0.050) | (0.059) |  | (0.066) | (0.066) | (0.079) | (0.079) |
| Constant | 5.992*** |  |  | 6.324*** | 5.587*** |  |  |
|  | (0.414) |  |  | (0.590) | (0.564) |  |  |
| Observations | 11407 | 11407 |  | 6347 | 6347 | 6347 | 6347 |
| ^a^ Dummy variables  Notes: Dependent variable = life satisfaction. BMI = body mass index, defined as height (in m) divided by weight (in kg) squared. Obesity = BMI≥30; overweight = 25≤BMI<30; normal weight = 18.5≤BMI<25. Standard errors are in parentheses. **p* < 0.1, ***p* < 0.05, ****p* < 0.01. | | | | | | | |

| **Table A4** OLS estimates of BMI on life satisfaction: SOEP 2002-2018 | | | | | | | | | |
| --- | --- | --- | --- | --- | --- | --- | --- | --- | --- |
|  | (1) | (2) | (3) | (4) | (5) | (6) | (7) | (8) | (9) |
|  | SWB | SWB | SWB | SWB | SWB | SWB | SWB | SWB | SWB |
| BMI (kg/m^2^) | -0.024** | -0.018* | -0.031*** | -0.041*** | -0.025** | -0.015* | -0.029*** | -0.024*** | -0.028*** |
|  | (0.010) | (0.011) | (0.010) | (0.014) | (0.012) | (0.009) | (0.007) | (0.007) | (0.008) |
| Ln(income) | 0.314*** | 0.377*** | 0.382*** | 0.349*** | 0.284*** | 0.312*** | 0.263*** | 0.389*** | 0.268*** |
|  | (0.069) | (0.065) | (0.052) | (0.067) | (0.059) | (0.053) | (0.051) | (0.051) | (0.058) |
| Age | -0.129*** | -0.198*** | -0.169*** | -0.135*** | -0.161*** | -0.119*** | -0.099*** | -0.074*** | -0.041* |
|  | (0.026) | (0.027) | (0.025) | (0.029) | (0.027) | (0.022) | (0.020) | (0.022) | (0.023) |
| Age squared | 0.001*** | 0.002*** | 0.002*** | 0.001*** | 0.002*** | 0.001*** | 0.001*** | 0.001** | 0.000 |
|  | (0.000) | (0.000) | (0.000) | (0.000) | (0.000) | (0.000) | (0.000) | (0.000) | (0.000) |
| Female^a^ | 0.248*** | 0.213** | 0.195** | 0.063 | 0.248*** | 0.167** | 0.128** | 0.303*** | 0.121 |
|  | (0.083) | (0.093) | (0.084) | (0.094) | (0.087) | (0.076) | (0.065) | (0.069) | (0.076) |
| Years of education | 0.016 | 0.020 | 0.032** | -0.001 | 0.020 | 0.013 | -0.000 | -0.018 | -0.008 |
|  | (0.014) | (0.014) | (0.013) | (0.015) | (0.014) | (0.013) | (0.011) | (0.012) | (0.013) |
| Married^a^ | 0.413*** | 0.438*** | 0.430*** | 0.448*** | 0.484*** | 0.341*** | 0.319*** | 0.383*** | 0.564*** |
|  | (0.084) | (0.091) | (0.086) | (0.087) | (0.092) | (0.078) | (0.067) | (0.071) | (0.073) |
| Number of children | -0.084** | -0.047 | 0.053 | 0.022 | 0.129*** | 0.046 | 0.069** | 0.054* | 0.033 |
|  | (0.036) | (0.041) | (0.042) | (0.049) | (0.050) | (0.049) | (0.029) | (0.032) | (0.034) |
| Homeowner^a^ | 0.252*** | 0.220*** | 0.232*** | 0.102 | 0.127* | 0.220*** | 0.169*** | 0.161** | 0.060 |
|  | (0.066) | (0.072) | (0.069) | (0.077) | (0.075) | (0.068) | (0.059) | (0.064) | (0.066) |
| Constant | 6.834*** | 7.044*** | 6.980*** | 7.482*** | 7.997*** | 6.916*** | 7.560*** | 6.163*** | 6.324*** |
|  | (0.845) | (0.751) | (0.675) | (0.833) | (0.756) | (0.673) | (0.556) | (0.584) | (0.590) |
| Observations | 5727 | 5305 | 5432 | 5137 | 4837 | 5143 | 7782 | 7032 | 6347 |
| ^a^ Dummy variables  Notes: Dependent variable = life satisfaction. BMI = body mass index, defined as height (in m) divided by weight (in kg) squared. Columns (1) through (9) use data from SOEP 2002, 2004, 2006, 2008, 2010, 2012, 2014, 2016 and 2018, respectively. Standard errors are in parentheses. *p < 0.1, **p < 0.05, ***p < 0.01. | | | | | | | | | |

| **Table A5** OLS estimates of overweight and obesity on life satisfaction: SOEP 2002-2018 | | | | | | | | | |
| --- | --- | --- | --- | --- | --- | --- | --- | --- | --- |
|  | (1) | (2) | (3) | (4) | (5) | (6) | (7) | (8) | (9) |
|  | SWB | SWB | SWB | SWB | SWB | SWB | SWB | SWB | SWB |
| Overweight^a^ | 0.002 | -0.153*** | -0.041 | -0.011 | -0.080 | -0.091* | -0.049 | -0.061 | -0.127** |
|  | (0.049) | (0.057) | (0.055) | (0.062) | (0.063) | (0.054) | (0.047) | (0.051) | (0.054) |
| Obesity^a^ | -0.136* | -0.228*** | -0.222*** | -0.275*** | -0.313*** | -0.254*** | -0.181*** | -0.222*** | -0.378*** |
|  | (0.074) | (0.078) | (0.078) | (0.086) | (0.084) | (0.070) | (0.061) | (0.062) | (0.067) |
| Ln(income) | 0.316*** | 0.302*** | 0.335*** | 0.284*** | 0.249*** | 0.241*** | 0.222*** | 0.270*** | 0.207*** |
|  | (0.041) | (0.044) | (0.038) | (0.046) | (0.044) | (0.036) | (0.035) | (0.036) | (0.041) |
| Age | -0.137*** | -0.153*** | -0.143*** | -0.119*** | -0.123*** | -0.101*** | -0.088*** | -0.075*** | -0.040** |
|  | (0.015) | (0.018) | (0.016) | (0.019) | (0.019) | (0.015) | (0.014) | (0.015) | (0.016) |
| Age squared | 0.001*** | 0.002*** | 0.002*** | 0.001*** | 0.001*** | 0.001*** | 0.001*** | 0.001*** | 0.000* |
|  | (0.000) | (0.000) | (0.000) | (0.000) | (0.000) | (0.000) | (0.000) | (0.000) | (0.000) |
| Female^a^ | 0.271*** | 0.186*** | 0.171*** | 0.155** | 0.233*** | 0.147*** | 0.113** | 0.223*** | 0.156*** |
|  | (0.052) | (0.062) | (0.057) | (0.063) | (0.064) | (0.051) | (0.048) | (0.048) | (0.054) |
| Years of education | 0.039*** | 0.032*** | 0.036*** | 0.027*** | 0.040*** | 0.027*** | 0.020** | 0.007 | 0.013 |
|  | (0.009) | (0.011) | (0.009) | (0.010) | (0.011) | (0.009) | (0.008) | (0.009) | (0.009) |
| Married^a^ | 0.342*** | 0.367*** | 0.338*** | 0.348*** | 0.398*** | 0.312*** | 0.307*** | 0.328*** | 0.480*** |
|  | (0.057) | (0.065) | (0.063) | (0.065) | (0.067) | (0.056) | (0.049) | (0.053) | (0.055) |
| Number of children | -0.012 | -0.025 | 0.066** | 0.039 | 0.065* | 0.054 | 0.068*** | 0.089*** | 0.047* |
|  | (0.026) | (0.029) | (0.032) | (0.035) | (0.037) | (0.034) | (0.022) | (0.023) | (0.026) |
| Homeowner^a^ | 0.296*** | 0.259*** | 0.273*** | 0.169*** | 0.114** | 0.228*** | 0.160*** | 0.193*** | 0.118** |
|  | (0.045) | (0.051) | (0.050) | (0.055) | (0.058) | (0.048) | (0.043) | (0.046) | (0.050) |
| Constant | 6.019*** | 6.426*** | 5.982*** | 6.271*** | 6.689*** | 6.771*** | 6.698*** | 6.438*** | 5.992*** |
|  | (0.414) | (0.424) | (0.395) | (0.454) | (0.479) | (0.380) | (0.344) | (0.369) | (0.414) |
| Observations | 11985 | 10793 | 10657 | 9725 | 9044 | 9391 | 14523 | 12844 | 11407 |
| ^a^ Dummy variables  Notes: Dependent variable = life satisfaction. BMI = body mass index, defined as height (in m) divided by weight (in kg) squared. Obesity= BMI≥30; overweight = 25≤BMI<30; normal weight = 18.5≤BMI<25. Columns (1) through (9) use data from SOEP 2002, 2004, 2006, 2008, 2010, 2012, 2014, 2016, and 2018, respectively. Standard errors are in parentheses. *p < 0.1, **p < 0.05, ***p < 0.01. | | | | | | | | | |

**Figure A1** Trends in the components of the intangible cost of overweight


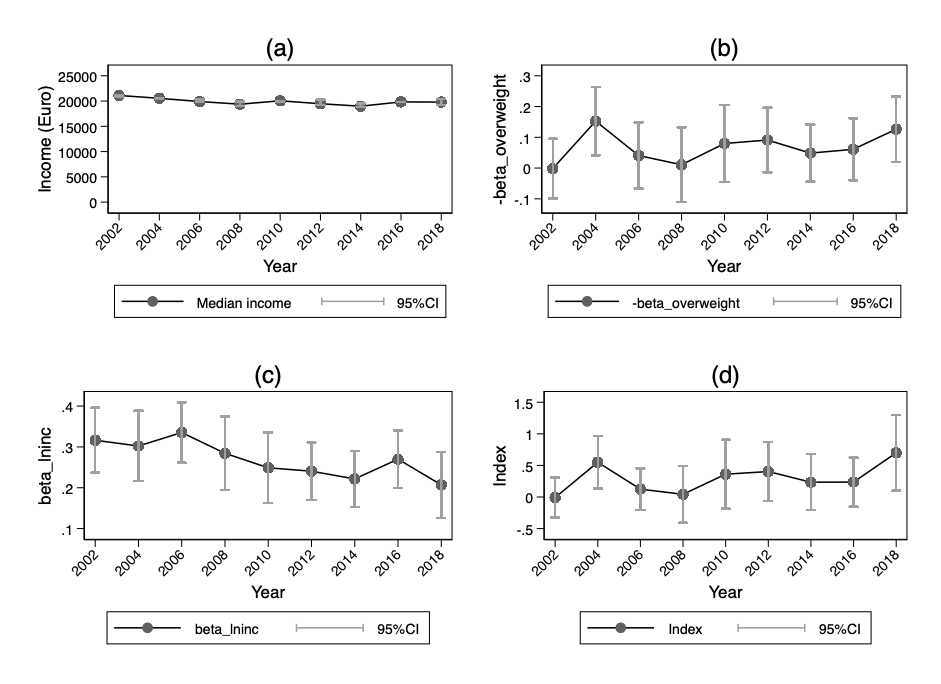


(a) median annual net income in 2018 euros. (b) and (c) the coefficients of overweight and income, respectively, based on equation 10. (d) the trend in the index, which denotes the negative division of the coefficient of overweight and income. The confidence interval is calculated using Fieller’s theorem (see Appendix Table A5 for regression results).

**Figure A2** Trends in the components of the intangible cost of obesity


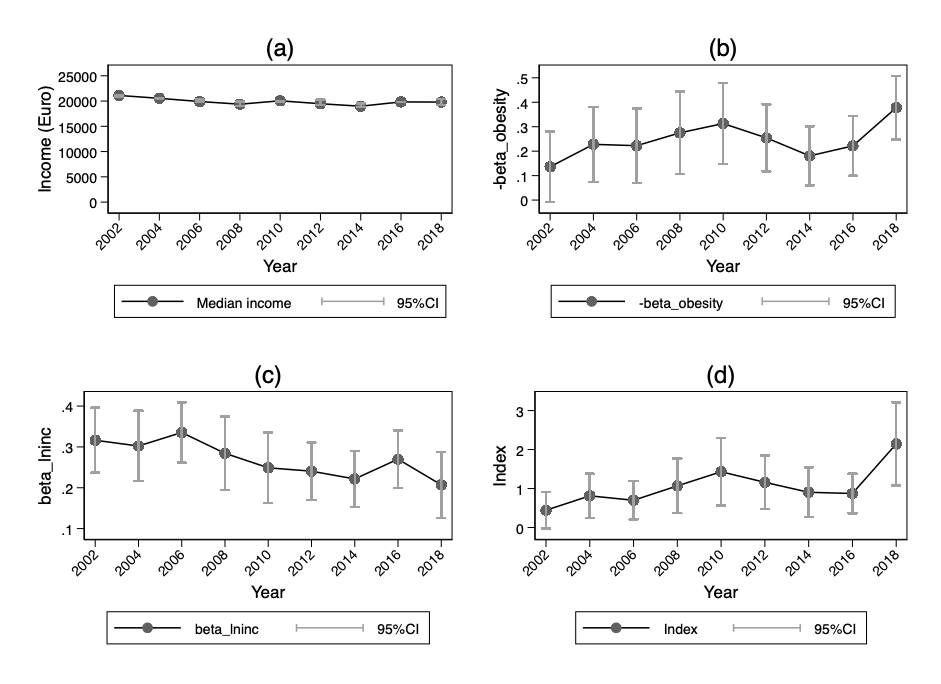


(a) median annual net income in 2018 euros. (b) and (c) the coefficients of obesity and income, respectively, based on equation 10. (d) the trend in the index, which denotes the negative division of the coefficient of obesity and income. The confidence interval is calculated using Fieller’s theorem (see Appendix Table A5 for regression results).

| **Table A6** OLS/ordered logit estimates of BMI on life satisfaction by different income levels: SOEP 2002-2018 | | | | | | | |
| --- | --- | --- | --- | --- | --- | --- | --- |
|  | OLS | | |  | Ordered logit | | |
|  | (1) | (2) | (3) |  | (5) | (6) | (7) |
|  | Low | Middle | High |  | Low | Middle | High |
| BMI (kg/m^2^) | -0.025*** | -0.021*** | -0.033*** | | -0.026*** | -0.023*** | -0.042*** |
|  | (0.006) | (0.006) | (0.005) |  | (0.005) | (0.006) | (0.006) |
| Ln(income) | 0.186*** | 0.617*** | 0.556*** |  | 0.161*** | 0.663*** | 0.758*** |
|  | (0.041) | (0.129) | (0.050) |  | (0.041) | (0.137) | (0.070) |
| Age | -0.137*** | -0.124*** | -0.123*** | | -0.138*** | -0.132*** | -0.160*** |
|  | (0.014) | (0.014) | (0.017) |  | (0.015) | (0.016) | (0.022) |
| Age squared | 0.001*** | 0.001*** | 0.001*** |  | 0.001*** | 0.001*** | 0.002*** |
|  | (0.000) | (0.000) | (0.000) |  | (0.000) | (0.000) | (0.000) |
| Female^a^ | 0.278*** | 0.188*** | 0.080* |  | 0.288*** | 0.238*** | 0.124** |
|  | (0.054) | (0.043) | (0.048) |  | (0.054) | (0.047) | (0.061) |
| Years of education | -0.023** | -0.001 | 0.002 |  | -0.022** | -0.002 | -0.001 |
|  | (0.011) | (0.009) | (0.006) |  | (0.011) | (0.010) | (0.008) |
| Married^a^ | 0.594*** | 0.351*** | 0.282*** |  | 0.574*** | 0.373*** | 0.361*** |
|  | (0.058) | (0.044) | (0.042) |  | (0.057) | (0.048) | (0.053) |
| Number of children | 0.024 | 0.019 | -0.010 |  | 0.023 | 0.029 | 0.010 |
|  | (0.029) | (0.024) | (0.019) |  | (0.029) | (0.028) | (0.025) |
| Homeowner^a^ | 0.213*** | 0.144*** | 0.118*** |  | 0.232*** | 0.144*** | 0.127*** |
|  | (0.045) | (0.039) | (0.036) |  | (0.047) | (0.044) | (0.046) |
| Constant | 8.557*** | 4.013*** | 5.107*** |  |  |  |  |
|  | (0.455) | (1.268) | (0.625) |  |  |  |  |
| Observations | 15274 | 17609 | 19859 |  | 15274 | 17609 | 19859 |
| ^a^ Dummy variables  Notes: BMI = body mass index, defined as height (in m) divided by weight (in kg) squared. Columns (1)-(3) report the OLS estimates for the low-, middle-, and high-income quartiles, respectively; columns (4)-(6) report the same estimates from the ordered logit model. *p < 0.1, **p < 0.05, ***p < 0.01. | | | | | | | |
